# The Dynamics of microRNA Transcriptome in Bovine Corpus Luteum during Its Formation, Function, and Regression

**DOI:** 10.3389/fgene.2017.00213

**Published:** 2017-12-15

**Authors:** Rreze M. Gecaj, Corina I. Schanzenbach, Benedikt Kirchner, Michael W. Pfaffl, Irmgard Riedmaier, Ry Y. Tweedie-Cullen, Bajram Berisha

**Affiliations:** ^1^Department of Animal Husbandry and Biotechnology, Faculty of Agriculture and Veterinary, University of Pristina, Pristina, Albania; ^2^Department of Animal Physiology & Immunology, Weihenstephan, Technical University of Munich, Munich, Germany; ^3^Department of Environmental Systems Science, ETH Zürich, Zurich, Switzerland; ^4^Dr. von Hauner Children's Hospital, Ludwig Maximillian Universität, München, Germany; ^5^Department of Physiology, TUM School of Life Sciences Weihenstephan, Technical University of Munich, Freising, Germany; ^6^Eurofins Medigenomix Forensik GmbH, Ebersberg, Germany; ^7^Department of Health Science and Technology, ETH Zürich, Zurich, Switzerland

**Keywords:** microRNA, small RNA Seq, bovine, corpus luteum, estrous cycle, SOTA

## Abstract

The formation, function, and subsequent regression of the ovarian corpus luteum (CL) are dynamic processes that enable ovary cyclical activity. Studies in whole ovary tissue have found microRNAs (miRNAs) to by critical for ovary function. However, relatively little is known about the role of miRNAs in the bovine CL. Utilizing small RNA next-generation sequencing we profiled miRNA transcriptome in bovine CL during the entire physiological estrous cycle, by sampling the CL on days: d 1–2, d 3–4, and d 5–7 (early CL, eCL), d 8–12 (mid CL, mCL), d 13–16 (late CL, lCL), and d > 18 (regressed CL, rCL). We characterized patterns of miRNAs abundance and identified 42 miRNAs that were consistent significantly different expressed (DE) in the eCL relative to their expression at each of the analyzed stages (mCL, lCL, and rCL). Out of these, bta-miR-210-3p, −2898, −96, −7-5p, −183-5p, −182, and −202 showed drastic up-regulation with a fold-change of ≥2.0 and adjusted *P* < 0.01 in the eCL, while bta-miR-146a was downregulated at lCL and rCL vs. the eCL. Another 24, 11, and 21 miRNAs were significantly DE only between individual comparisons, eCL vs. the mCL, lCL, and rCL, respectively. Irrespective of cycle stage two miRNAs, bta-miR-21-5p and bta-miR-143 were identified as the most abundant miRNAs species and show opposing expression abundance. Whilst bta-miR-21-5p peaked in number of reads in the eCL and was significantly downregulated in the mCL and lCL, bta-miR-143 reached its peak in the rCL and is significantly downregulated in the eCL. MiRNAs with significant DE in at least one cycle stage (CL class) were further grouped into eight distinct clusters by the self-organizing tree algorithm (SOTA). Half of the clusters contain miRNAs with low-expression, whilst the other half contain miRNAs with high-expression levels during eCL. Prediction analysis for significantly DE miRNAs resulted in target genes involved with CL formation, functionalization and CL regression. This study is the most comprehensive profiling of miRNA transcriptome in bovine CL covering the entire estrous cycle and provides a compact database for further functional validation and biomarker identification relevant for CL viability and fertility.

## Introduction

In mammalian reproduction the corpus lutem (CL) is a pivotal gland, primarily because of its role in regulating the length of estrous cycle. The CL development is completed rapidly after the egg ovulation from the follicular remnants and its structural features remain stable, it instead secretes the hormone progesterone (P4), at levels required for ovarian cyclical activity and maintenance of pregnancy when it occurs (Reynolds and Redmer, 1999; Davis and Rueda, 2002; Stocco et al., 2007; Rekawiecki et al., 2008). As a highly dynamic tissue, exhibiting periodical growth, functionalization and regression, the CL is an ideal example for the study of diverse cellular processes that occur in it: cell proliferation and apoptosis, immune system activation, down regulation of functional proteins, and cellular matrix remodeling events (Davis et al., 2003; Schams and Berisha, 2004; Sugino and Okuda, 2007; Yoshioka et al., 2013).

Over the past few years many studies have helped elucidate the regulatory events underlying bovine CL development and their function/s at the transcription level. These studies have highlighted and further reinforced the importance of several classes of growth factors as key regulators of CL formation (angiogenesis), function (Berisha et al., 2000b, 2002, 2004), and CL regression (Reynolds et al., 2000; Neuvians et al., 2004; Berisha et al., 2008, 2010, 2016, 2017; Miyamoto et al., 2010; Nishimura and Okuda, 2010). Although the well-orchestrated transcriptional changes in bovine ovary and CL are described in considerable details (Berisha et al., 2000a,b, 2002, 2004, 2008, 2010, 2016, 2017; Neuvians et al., 2003, 2004; Kliem et al., 2007; Shimizu et al., 2007; Sarkar et al., 2011), very little in regard to the post-transcriptional regulation has been addressed. In particular the role of post-transcriptional regulators such as miRNAs that act locally and in a cell-type specific manner is not clear yet (Wienholds and Plasterk, 2005). MiRNAs are short non-coding and single-stranded nucleic acids that regulate the translation of mRNA transcripts during tissue development and differentiation (Lee et al., 1993; Bartel, 2004; Kloosterman and Plasterk, 2006; Stefani and Slack, 2008). They regulate the abundance of gene protein products by inhibiting translation or by degrading the targeted mRNA itself (Shivdasani, 2006). Gene expression in the CL as an endocrine gland is subjected to dynamic regulations that are likely influenced by miRNAs from development through luteolysis. Indeed, there is experimental evidence supporting the involvement of miRNAs in the formation of CL. Using a *Dicer1* (*an* RNase III enzyme required for processing miRNAs) hypomorphic mouse line (*Dicer*^*d*/*d*^), Otsuka et al. (2008) demonstrated that the females form this line were infertile. The observed infertility was shown to be due to the altered CL development resulting mainly from an impaired vascular network. The mechanism for this phenotype was linked to the up-regulation of the anti-angiogenic transcription factors: tissue inhibitor of metalloproteinase 1 (*TIMP1*) and platelet factor 4 (*PF4*). Both transcription factors were shown to be predicted targets of the miRNAs: 17-5p and let-7b, and indeed their expression were restored to baseline by the injection of these two miRNAs. In bovine the preovulatory luteinizing hormone (LH) surge is the key event leading to major cellular phenotype changes in follicles and changes in CL transcriptome (Sood et al., 2006; Christenson et al., 2013). The ongoing cellular alterations suggest there might be stage specific populations of miRNAs in the developing, fully functional and then regressing CL, which could be induced by the secretion of other hormones e.g., P4 (Yao et al., 2010).

The published data involving miRNAs in the regulation of CL function in large farm animals are rather limited and conducted on either whole ovary tissue (Tripurani et al., 2010; Li et al., 2011; Sontakke et al., 2014) or in the CL from a specific stage (Maalouf et al., 2014, 2016), consequently a complete and detailed picture is missing.

Therefore the objective of this study was to analyze the miRNA transcriptome dynamics at different timely defined CL classes (days). Specifically, we sequenced miRNAs transcriptome during early CL formation (C1: d 1–2, C3: d 3–4, and C5: d 5–7), mid CL function (C8: d 8–12), late CL function (C13: d 13–16) and during the CL regression (C18: d > 18). We identified 42 miRNAs that were consistently significant DE across the analyzed CL classes. Further 24, 11, and 21 miRs showed significant DE only between individual comparisons eCL vs. mCL, lCL, and rCL, respectively.

Results from the present study will create knowledge on miRNA transcriptome in the field of animal reproduction and provide a fertile ground for any further functional validation studies or biomarker identification beyond reproduction.

## Materials and methods

### Animals and tissue collection

The corpora lutea were collected within 20 min post the routine slaughter from German Fleckvieh Cattle at local abattoirs. The isolated luteal tissue was immediately frozen in liquid nitrogen and stored at −80°C until used in experiments.

The stage of the estrous cycle was determined by macroscopic examination as previously described (Berisha et al., 2016, 2017). In brief: macroscopic evaluation of the whole ovaries by assessing their color, consistency and of the uterus by assessing its color, consistency and the mucus were considered for the determination of CL stages. According to that the CL were assigned into the following classes (days): early CL (eCL, C1: d 1–2, C3: d 3–4, C5: d 5–7), mid CL (mCL, C8: d 8–12), late CL (lCL, C13: d 13–16) and regressed CL (rCL, C18: d >18). A total of 18 corpora lutea, each from individual animals, were included in the study. Three corpora lutea were used for each analyzed CL classes (C1, C3, C5, C8, C13, and C18) and subsequent small RNA-Seq.

### RNA extraction and MicroRNA quantification

For the extraction of total RNA and subsequent purification of miRNA, slices from frozen CL were cut and processed using the miRNeasy Mini Kit (Qiagen, Germany) according to the manufacture's protocol. RNA was quantified using a Nanodrop spectrophotometer (Thermo Fisher Scientific, Germany), purity was determined based on the optical density ratio and mRNA quality was assessed on a Bioanalyzer 2100 (Agilent Technologies, Germany) using the RNA 6000 Nano Assay. Only CLs with a RIN value higher than 8 were considered (Mueller and Schroeder, 2004; Fleige and Pfaffl, 2006). In order to determine the percentage of small RNA relative to total RNA, samples were further analyzed using the Small RNA Kit (Agilent Technologies, Germany), which allows the quantification of small RNA ranging in size between 6 and 150 nts.

### Preparation of library for small RNA sequencing

Library preparation was performed using the NEBNext Multiplex Small RNA Library Prep Set for Illumina (New England BioLabs Inc., USA). A total of 200 ng of purified RNA was used as starting material. This was converted into barcoded cDNA according to Protocol E7330 (New England BioLabs Inc., USA) and sequenced on the Illumina HiSeq2500 platform (Illumina Inc., USA). Briefly the steps are: 3′-adaper ligation, followed by reverse transcription primer hybridization (to prevent adaptor-dimer formation), 5′-adapter ligation, a reverse transcription reaction and PCR enrichment (Spornraft et al., 2014). The PCR products were then purified with the MinElute PCR Purification Kit (Qiagen, Germany) and their length checked on the Bioanalyzer 2100 (Agilent, Germany) using the DNA 1000 Kit (Agilent, Germany). The cDNA constructs were size selected by loading the PCR products on a 4% agarose gel. Bands corresponding to products of 140–160 bp in length were cut (Supplementary Figure 1) and processed by the MinElute Gel Extraction Kit (Qiagen, Germany). The size of the cDNA library was validated using the High Sensitivity DNA Kit (Agilent Technologies, Germany) (Supplementary Figure 1). Library constructs were then sequenced on the HiSeq2500 Illumina platform.

### Sequencing quality and mapping

The sequencing data were processed using in-house tools (Spornraft et al., 2014, 2015; Buschmann et al., 2016; Schanzenbach et al., 2017). The trimming program, Btrim (Kong, 2011), was used to trim the adaptor sequence from the 3′ ends and all reads without detectable adaptor sequences were removed from the dataset. Next the length distribution and the base call accuracy, represented by the phred quality scores (Q score), were calculated using the quality control software FastQC (Babraham Bioinformatics, UK, Version 0.10.1). In order to prevent bias and false-positive mapping by degenerated RNA or other small RNA material, all reads shorter than16 nts were removed from the dataset. The remaining reads were filtered for bovine rRNA, tRNA, snRNA, and snoRNA sequences by mapping them to the RNAcentral database (RNAcentral Consortium, 2015). The filtered dataset was aligned to the most recent miRBase database for mature bovine miRNAs (release 21) (Kozomara and Griffiths-Jones, 2014) by using the BowTie short read aligner (Langmead et al., 2009). Default parameters and settings were used except for the “best” alignment algorithm, which was adapted by allowing only a single mismatch across the whole sequence. All aligned reads were subsequently sorted and indexed by SAMtools (Li et al., 2009), the read counts were then generated by retrieving the sum of hits per miRNA sequence.

### Reverse transcription and real-time PCR

The miScript PCR assays from Qiagen were used for the validation of the NGS results. The regulated miRNAs were quantified from 100 ng of the total RNA (*n* = 18 animals) and reversed transcribed using the 5x miScript HighSpec buffer. The RT-PCR was carried out in a Rotor—Disc 100 on the Rotor-Gene Q, Real-Time Detection System. For validation the following miScript assays were used: Bt_mir-210_1, Bt_mir-96_1, Bt_mir-146a_1, Bt_mir-182_1, Bt_mir-183_1, Bt_mir-2898_1, Bt_mir-7_1, Bt_mir-202_1, Bt_mir-21-5p_1, and Bt_mir-143_1.

RNA pools lacking an RT enzyme from different CL samples as well as an additional water control sample were included in each qPCR run. The expression data were normalized using the Hs_RNU6-2_1 gene and measurements were undertaken following the Pfaffl method (Pfaffl, 2001).

### Statistical analysis

Statistical analysis on read counts, representing the frequency of the sequenced miRNAs, was performed using the DESeq2 R script (v.3.1.2). This methodology is based on a negative binomial distribution, linking the mean and variance by local regression (Love et al., 2014) and determines the adjusted *P*-value (FDR; Benjamini and Hochberg adjustment) allowing for across sample comparison. A read count noise-cut-off >50 was applied for all miRNAs identified as DE. Distribution of significantly DE miRNAs across the estrous cycle was assessed by the web application Bio Venn (Hulsen et al., 2008).

### SOTA: self-organizing tree algorithm and unsupervised hierarchical clustering (HCL)

The SOTA and HCL were used to search for correlating miRNA expression patterns (Dopazo and Carazo, 1997; Herrero et al., 2001). All miRNAs with average read counts >50 and at least one cycle stage of significant DE were included in the search. The mean centered expression was calculated as follows: mean read values of replicate CL samples (*n* = 3) for each cycle stage was assessed and log2 transformed. In the next step, the mean value of all samples was subtracted from the cycle stage specific value. HCL and SOTA were performed using the MeV_4 Software (v. 4.9.0) (Saeed et al., 2003) with default settings. For SOTA a limit of seven cycles was applied allowing for a total of eight generated clusters.

### Micro RNA target identification and pathway analysis

Identification of putative miRNAs targets for DE miRNAs common to all conditions (# 42miRNAs) was performed using the miRTarBase release 6.0 (Chou et al., 2016) and TargetScan release 7.0 (http://www.targetscan.org). We selected miRNA-target interactions (MTIs) that were experimentally validated in human and rodents by RT-PCR, reporter assay or Western blot as detailed in http://mirtarbase.mbc.nctu.edu.tw/ and did an *in silico* prediction of their targets within the bovine genome using the TargetScan (v7.0).

The open access DIANA miRPath v.30 software (Vlachos et al., 2015) was used to generate pathways enriched for genes targeted by miRNAs identified as DE between individual comparisons: eCL vs. mCL, lCL, and rCL. The latest version of DIANA microT-CDS (v5.0) with a microT threshold of 0.8 and a false discovery rate (FDR) correction for the threshold *p* < 0.05 were applied. To exclude any pathways containing only a few targeted gene nodes the modified Fisher's exact test was applied. DIANA-miRPath database and the miRTarBase consist of validation studies that have been mainly performed on human or rodents and less in bovine, however miRNAs are highly functionally conserved between species and their nomenclature is based on seed sequence similarities (Friedman et al., 2009).

## Results

### Small RNA sequence quality assessment

Each sample, representing one animal, was analyzed for the presence of high quality RNA reads. The assessment of the quality of sequenced samples was performed using the fastqc algorithm. Both, per base and per sequence, quality scores were considered. In addition, the mean sequence quality (Phred Score) for all 18 sequenced CL samples was evaluated. Overall, 98% of all reads showed a quality (Q) score of 37 (Supplementary Figure 2). It is known that base calls with a Q-score higher than 30, contain one incorrect base call in 1,000 and therefore have an error probability of 0.001 (Ewing and Green, 1998). According to the HiSeqIlumina platform, if more than 90% of reads have an average Q-score of 30 or greater, they are considered reads of high quality (Illumina, 2011). The total sequence length showed a clear pattern in its distribution with a peak at 22 nts (Supplementary Figure 2), which corresponds to the known length of miRNA and the mean GC content per sequence was around 47% (Supplementary Figure 2), ensuring thus non-biased annotation of the sequencing data, due to the ubiquitously genome presence of GC regions.

### Evaluation of small RNA sequencing data

The mean number of sequence reads across the 18 small RNA Seq analyses were 15,244,516 ± 4,070,073 (*SD*) (Supplementary Table 1). In total 9,211,818 ± 2,048,812 (*SD*) sequences passed the trimming threshold, whilst 6,031,779 ± 2,039,033 (*SD*) did not and were excluded from any further analysis. The sequences that passed trimming were further mapped to the RNAcentral database to remove all small RNAs, which were not microRNA in nature (RNAcentral Consortium, 2015). The resulting number of filtered sequences for each sample was 3,683,423 ± 653,417 (*SD*), while a mean of 5,730,324 ± 1,438,850 (*SD*) sequences did not map to RNAcentral. These sequences were annotated by mapping them to the bovine mirbase (version 21, Kozomara and Griffiths-Jones, 2014). This allowed a mean 898,679 ± 289,266 (*SD*) sequences across the CL samples to be mapped onto annotated miRNAs, making up 5.1 ± 2.9% (*SD*) of the total read counts (Supplementary Table 1).

**Figure 1:**
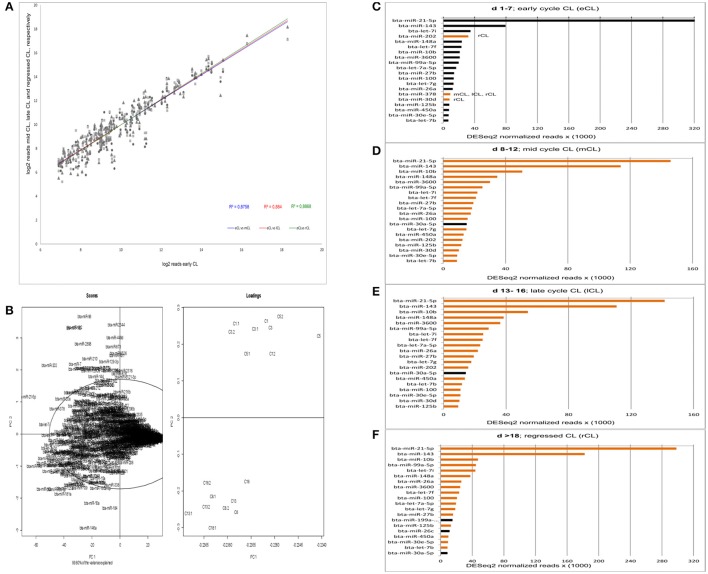
Acquisition of read correlation, general data behavior and miRNAs abundance. **(A)** Correlation of NGS normalized reads between the comparisons eCL vs. mCL, lCL, and rCL. **(B)** Scores and loadings plot (SL-plot) showing the specific clustering of miRNAs from six different CL classes. The normalized reads are clustered using DESeq2. Each CL class consists of three CL from individual animals (e.g., C1, C1.1, andC1.2 belong to the CL class C1). **(C–F)** Top 20 most abundant miRNAs at each CL class. Black bars at either mCL, lCL, or rCL represent miRNAs abundant at these CL classes and absent in the eCL. The orange bars at eCL represent miRNAs abundant at this CL class and absent in the subsequent CL classes.

The overall numbers of read were well-correlated between eCL and the three other stages (Figure 1A). Yet, by the acquisition of overall data behavior as can be seen in Figure 1B, we found that the eCL was clearly separated from mCL, lCL, and rCL. Performing principal components of scores and loadings (SL-plot), two distinct and significantly different clusters were identified, one corresponding to the eCL class (Figure 1B, loadings, top right) and the other to the mCL, lCL, and rCL classes (Figure 1B, loadings bottom right). The two clusters were separated by principal component 1 (PC1) which explained 99.8% of the total data variance and clearly differentiated eCL from mCL, lCL, and rCL.

PC1 is determined by the highly abundant and significantly DE miRs: miR-21-5p (Figure 1B, scores upper quadrant and Figure 1C) and miR-143 (Figure 1B, scores lower quadrant and Figure 1F). The PC2 is mainly determined by less abundant, hence significant DE miRs: miR-146a (Table 1, significantly downregulated in mCL, lCL, and rCL vs. the eCL,) and miR-96 (Table 1, significantly upregulated in mCL, lCL, and rCL vs. the eCL). Nevertheless the cumulative effect of other DE miRNAs (Table 1) also counts for the cluster separation of our data.

**Table 1:** Differentially expressed bovine miRNAs throughout the CL lifespan.

**mir-ID**	**Reads**				**Fold-change NGS**			**Adjusted *P*-value (‡)**		
	**eCL**	**mCL**	**lCL**	**rCL**	**eCL vs. mCL**	**eCL vs. lCL**	**eCL vs. rCL**	**eCL vs. mCL**	**eCL vs. lCL**	**eCL vs. rCL**
181a-5p	2,254	6,492	6,222	4,998	−1.5	−1.4	−1.1	9.48E-21	7.95E-19	7.17E-12
146a	88	255	430	566	−1.5	−2.2	−2.6	2.32E-08	1.28E-17	3.32E-24
7-5p	1,567	326	526	667	2.2	1.5	1.2	2.38E-22	2.34E-11	2.49E-07
210-3p	306	55	90	110	2.4	1.7	1.4	2.26E-16	8.07E-09	1.10E-06
10b	21,027	50,773	53,914	47,295	−1.2	−1.3	−1.1	1.30E-07	1.67E-08	1.12E-06
125a	1,890	4,413	4,297	4,470	−1.2	−1.2	−1.2	5.36E-07	9.92E-07	2.90E-07
2898	755	232	141	118	1.6	2.3	2.5	2.68E-06	2.34E-11	3.16E-13
296-3p	232	88	81	56	1.4	1.5	1.9	4.72E-06	8.19E-07	6.42E-11
96	819	80	158	129	2.8	2.1	2.3	1.76E-08	3.15E-05	3.74E-06
7857	80	21	31	24	1.8	1.3	1.7	2.64E-08	5.42E-05	7.26E-07
183-5p	2,453	339	637	456	2.5	1.8	2.2	1.45E-08	6.17E-05	9.52E-07
30c-5p	535	1,338	1,261	955	−1.3	−1.2	−0.8	2.35E-10	3.44E-09	9.55E-05
30a-5p	4,960	15,067	14,205	8,631	−1.6	−1.5	−0.8	1.16E-15	5.88E-14	1.40E-04
let-7e	273	532	532	510	−0.9	−0.9	−0.9	8.02E-05	5.71E-05	1.95E-04
6119-5p	353	187	213	212	0.9	0.7	0.7	2.00E-06	1.69E-04	1.79E-04
2478	481	211	135	114	1.2	1.7	2.0	4.19E-04	3.45E-08	3.88E-10
182	2,348	283	631	456	2.6	1.7	2.1	7.10E-08	4.47E-04	2.35E-05
1307	332	184	184	139	0.8	0.8	1.2	4.98E-04	3.48E-04	1.68E-07
10a	72	248	199	228	−1.6	−1.4	−1.5	7.25E-05	8.90E-04	1.79E-04
17-5p	1,271	644	543	715	1.0	1.2	0.8	5.48E-04	3.03E-04	3.33E-03
433	321	114	175	145	1.4	0.9	1.1	1.47E-06	5.58E-03	2.14E-04
26a	11,965	17,726	21,948	26,243	−0.6	−0.9	−1.1	6.92E-03	1.06E-05	6.71E-09
1260b	269	128	64	67	1.0	1.9	1.9	7.11E-03	1.54E-07	3.65E-07
148a-3p	23,583	34,733	38,414	37,599	−0.6	−0.7	−0.7	6.75E-03	3.12E-04	6.75E-04
26b	1,924	2,726	3,409	3,169	−0.5	−0.8	−0.7	1.24E-02	1.27E-05	1.79E-04
202	31,881	12,366	15,810	5,617	1.3	1.0	2.3	9.04E-04	1.33E-02	3.88E-10
381	1,941	794	916	1,151	1.2	1.0	0.7	1.91E-05	3.03E-04	1.58E-02
139	328	927	884	561	−1.4	−1.4	−0.8	1.47E-06	3.60E-06	1.66E-02
191	2,069	2,776	2,609	2,728	−0.4	−0.3	−0.4	1.79E-03	1.49E-02	3.13E-03
22-3p	797	1,280	1,443	1,082	−0.7	−0.8	−0.4	2.20E-04	1.54E-06	2.02E-02
450b	1,068	1,804	2,019	1,467	−0.7	−0.9	−0.5	7.80E-05	8.42E-07	2.14E-02
107	730	1,048	1,234	1,128	−0.5	−0.7	−0.6	1.82E-02	2.56E-04	3.17E-03
26c	5,013	7,232	9,167	11,279	−0.5	−0.9	−1.1	2.34E-02	5.71E-05	5.65E-08
132	744	338	171	182	1.1	1.9	1.8	2.61E-02	2.65E-05	6.17E-05
29c	39	90	105	61	−1.2	−1.4	−0.6	6.34E-06	6.14E-08	2.68E-02
20a	5,053	3,193	2,297	2,890	0.6	1.1	0.8	2.33E-02	3.07E-05	4.08E-03
493	571	373	254	331	0.6	1.1	0.8	2.97E-02	1.20E-05	4.12E-03
1248	95	199	211	158	−1.0	−1.1	−0.7	1.58E-03	3.92E-04	3.27E-02
450a	7,245	13,107	13,603	9,798	−0.8	−0.9	−0.4	1.61E-05	3.38E-06	3.76E-02
92b	218	125	134	88	0.8	0.7	1.2	1.19E-02	2.82E-02	2.42E-05
574	687	458	452	428	0.6	0.6	0.7	2.08E-02	1.49E-02	5.20E-03
15a	45	74	119	75	−0.7	−1.3	−0.7	4.52E-02	3.97E-05	4.09E-02

*A total of 42 miRNAs were common to all comparisons. The read counts for each miRNA are given as the base mean of three CLs replicates. The mean read count ratio between either early CL (eCL) and mid CL (mCL), or late CL (lCL) and regressed CL (rCL), is shown as the fold change (FC). MiRNAs are listed in the order of their adjusted P-value (Padj). Down-regulated miRs: FC < 1. Up-regulated miRs: FC >1. The fold changes and adjusted P-values were calculated using DESeq2*.

‡ *FDR, Benjamini and Hochberg correction*.

### Identification of miRNAs in the CL at different cycle stages

Over all CL samples we found 439 miRNAs with more than one read in average, of them 205 account for 99.7% of the total reads. Furthermore, 8.65% of the annotated miRNAs had 1,000–250,000 reads, 17.2% had between 50 and 1,000 reads and 74.15% show fewer than 50 reads (Supplementary Table 2).

The expression level of the most abundant miRNAs was determined by their number of read counts, which reflects how frequently they were sequenced. The top 20 most abundantly expressed miRNAs for each cycle stage are shown in Figures 1C–F. Interestingly, we found that two miRNAs, bta-miR-21-5p and bta-miR-143, show constantly greater expression and account for more than 40% of the total number of reads in the CL samples across the cycle. A decrease in number of read counts, and hence abundance, was seen for bta-miR-21-5p from early to mid, late and the subsequent regression whilst the opposite was observed in the case of bta-miR-143 (Figures 1C–F). Several other miRNAs were observed that vary in expression across the CL cycle. For example, bta-miR-202 was highly expressed during the early CL, however its number of reads decreases in the subsequent CL classes and in the rCL is not among the top 20 most abundantly expressed miRNAs (Figure 1D). Conversely, bta-miR-30a-5p is highly abundant in mCL, lCL, and rCL, but not in the eCL (Figures 1B–D). Notable, the list of top 20 most abundantly expressed miRNAs shows similar expression pattern in the eCL, mCL, and lCL, while greater discrepancy is observed between eCL and rCL (Figures 1A,D, bars with either black or orange color).

### Significantly DE miRNAs in the CL during estrous cycle

**Figure 2:**
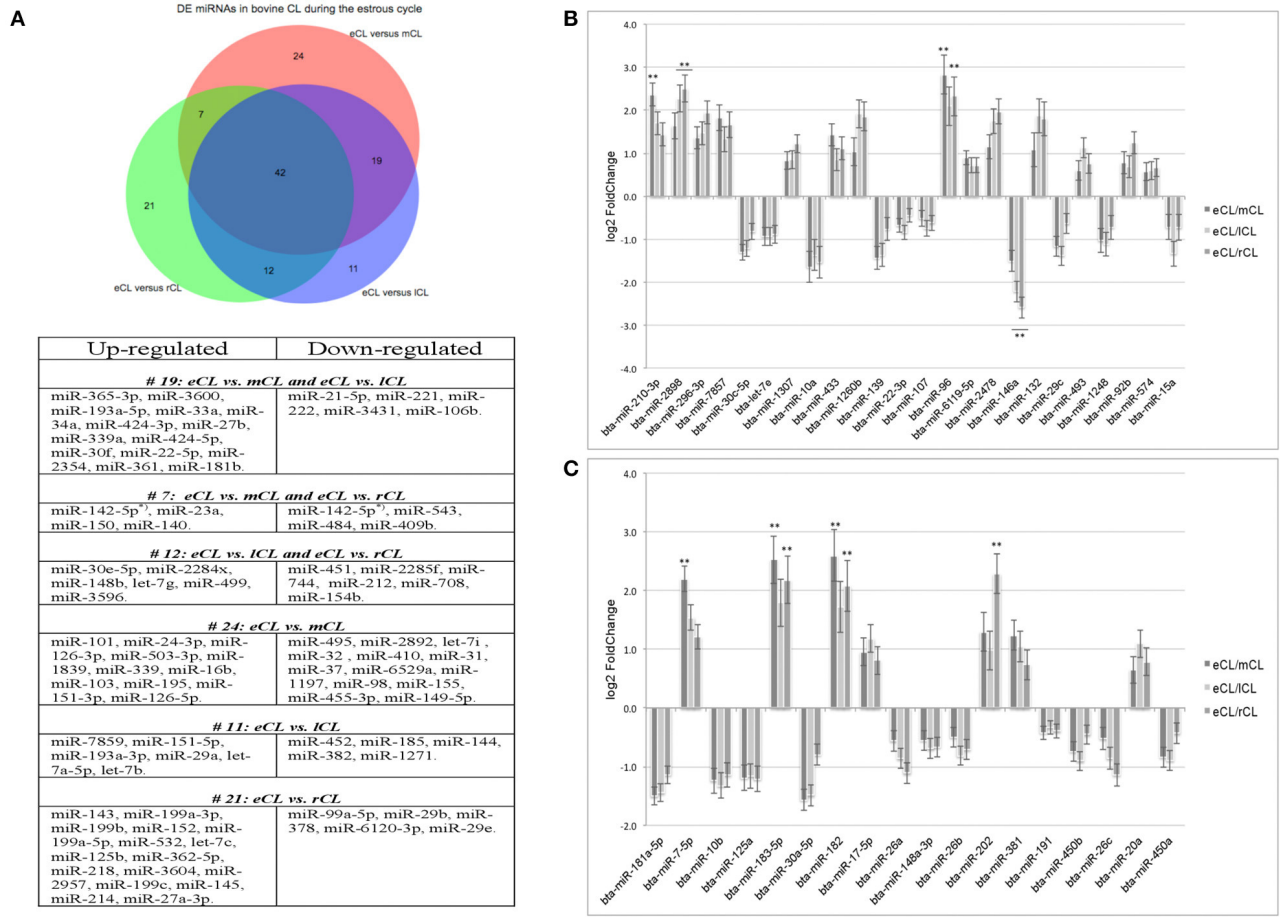
Distribution of differentially expressed (DE) miRNAs across all comparisons and log2 fold-changes of miRNAs consistently DE in all three CL classes vs. the eCL. **(A)** Venn diagram showing the overall number of DE miRNAs and their relation to each of the analyzed CL classes being: early CL (eCL) vs. mid CL (mCL), late CL (lCL), and regressed CL (rCL), respectively. **(B)** log2 fold-changes of miRNAs with <1,000 reads in the eCL. **(C)** log2 fold-changes of miRNAs with >1,000 reads in the eCL. The fold changes and adjusted *P*-values were calculated using DESeq2 (^**^adjusted *P* < 0.01). Error bars are shown as log fold-change standard error (lfcSE). Note: only miRNAs with FC > 2.0 are marked with asterisk; adjusted *P*-values for miRNAs with FC < 2 are presented in Table 1.

Using the DESeq2 algorithm (Anders and Huber, 2010) we identified differentially expressed miRNAs at the comparisons between stages, namely eCL to mCL, lCL, and to rCL. In the comparison between eCL and mCL samples we identified 92 DE miRNAs (Supplementary Table 3A). Of these, 42 miRNAs were also found to be DE in two other CL classes, lCL and rCL, vs. the eCL (Figures 2A–C, Table 1).

In contrast, 24 miRNAs were DE regulated only between individual comparison of eCL and mCL, while a total of 19 miRNAs were common to comparison of eCL to mCL and eCL to lCL. Other 7miRNAs were common to the comparisons of eCL to mCL and eCL to rCL (Supplementary Tables 3A, 4A). The complete list of DE miRNAs in eCL vs. the other cycle stages as well as all common or unique miRNAs to a given comparison can be accessed as supplementary information (Supplementary Tables 3A–C, 4A,B).

Overall, 21 miRNAs out of the 42 miRNAs significantly DE across all comparisons were seen to be upregulated in the mid, late and regressed CL stages relative to their expression in the eCL (Figures 2B,C, Table 1, FC >1). The remaining 21 miRNAs were downregulated for the same comparison (Figures 2B,C, Table 1, FC < 1). Yet, seven of them: bta-miR-210, −2898, −96, −7-5p, −183-5p, −182, and −202 show a fold change (FC) up-regulation ≥2.0 with an adjusted *P* < 0.01 in at least one of the three CL classes (mCL, lCL, and rCL) vs. the eCL. In contrast, only one miRNA bta-miR-146a was downregulated with a FC ≥ 2.0 and adjusted *P* < 0.01 in the lCL and rCL vs. the eCL. MiRNAs with FC ≥ 2.0 and adjusted *P* < 0.01 are indicated with asterisk (Figures 2B,C, Table 1). Other significantly regulated miRNAs with a FC below 2.0 can be found in the Table 1. MiRNAs upregulated in eCL vs. the three other stages are in bold only with a positive FC, while miRNAs downregulated in the eCL vs. all three other stages are indicated with a negative FC.

As the cycle progresses the number of miRNAs found to be significantly DE was slightly decreased and 84 miRNAs were regulated between eCL early and lCL (Supplementary Table 3B). Of these 11 are unique as beeing regulated only in eCL vs. lCL and not in other comparisons (Figure 2A, Supplementary Table 4B). We next compared eCL to rCL and identified a total of 82 DE miRNAs (Supplementary Table 3C), of which 21 are unique to this comparison (Figure 2A, Supplementary Table 4B). Details of all regulated miRNAs with their respective log2FC and their adjusted *P*-values across all comparisons can be accessed in the supplementary data (Supplementary Tables 3A–C), while the distribution of common and unique miRNAs DE between the eCL and other CL classes is provided as Supplementary Tables 4A,B.

### RT-qPCR and miRNA validation

Eight miRNAs (bta-miR-210, -96, -7, -182, -183, -2898, -146a, and -202) that were found to be DE regulated across the different CL classes as well as two miRNAs (bta-miR-21-5p and bta-miR-143) identified as the most abundantly expressed miRNA species are chosen for RT-qPCR validation.

For six miRNAs (bta-miR-210, −96, −182, −183, −146a, and −202) out of the eight selected miRNAs the qPCR results were consistent with those from the NGS and all of them are upregulated in the eCL vs. the three other stages. Statistical significance was obtained and indicated in Tables 2, 3, Supplementary Figure 3.

**Table 2:** Fold-change regulation of DE miRNAs during the estrous cycle obtained by RT-qPCR and NGS.

**miR-ID**	**qPCR**	**NGS**	**qPCR**	**NGS**	**qPCR**	**NGS**
	**eCL/mCL**		**eCL/lCL**		**eCL/ CL**	
bta-miR-210	**0.3**	2.4	0.8	1.7	0.6	1.4
bta-miR-96	**0.2**	2.8	**0.3**	2.1	**0.1**	2.3
bta-miR-7	0.5	2.2	1.2	1.5	0.7	1.2
bta-miR-182	**0.2**	2.6	**0.5**	1.7	**0.2**	2.1
bta-miR-183	**0.3**	2.5	0.5	1.8	**0.3**	2.2
bta-miR-2898	1.0	1.6	0.7	2.3	0.7	2.5
bta-miR-146a	−2.0	−1.5	−**4.4**	−2.2	−**4.3**	−2.6
bta-miR-202	0.6	1.3	1.2	1.0	**0.2**	2.3
bta-miR-21-5p	**0.48**	1.08	0.86	1.12	1.07	0.13
bta-miR-143	−**2.41**	−0.51	−**3.85**	−0.48	−**3.72**	−1.16

*The RT-qPCR fold change regulation was calculated using the 2^−ddCt^. Significance between the CL classes was assessed by Student's t-test on the normalized Ct values (dCt-Value). Significant regulation (p < 0.05) are indicated in bold. Detailed information for individual miRNA level of significance regulation is provided in the Supplementary Figure 3. NGS normalized reads are significantly regulated in all three comparisons and are detailed on Table 1*.

**Table 3:** Selected candidate genes related to CL development, function, and regression that are targeted by DE miRNAs common to all: eCL vs. mCL, lCL, and rCL.

**Functional category**	**Genes^(a)^**	**Differentially expressed miRNAs**
Cell proliferation	PTEN	**miR-17-5p**, miR-107, miR-29a-5p, miR-182-5p, miR-29c-3p, miR-20a
	CDKN1B	**miR-181a-5p**, **miR-182-5p**, miR-30a-5p, miR-148a-3p
	CDKN1A	**miR-17-5p**, miR-125a-5p, miR-146a-5p, miR-1260b, miR-574, miR-182-5p, miR-20a-5p
	Cdk6	miR-191-5p
CL formation (angiogenesis)	VEGFA	miR-125a-5p, **miR-17-5p**, miR-107, miR-15a-5p, miR-29c-3p, **miR-20a**
	HIF1A	miR-210-3p, miR-22-3p, **miR-17-5p**, miR-107, miR-20a
	FGF2	miR-107, miR-15a-5p
	FGF9	miR-26a-5p, miR-433-3p, miR-182
	PGR	**miR-181a-5p**
	ANGPT1	miR-15a-5p
	ANGPT2	miR-181a-5p
	IGF1R	bta-let-7e-5p, miR-139
	S1PR1	miR-148a-3p
	EDN2	miR-26b-5p
ECM- remodeling	FLT1	miR-20a
	FGFRL1	miR-210-3p
	THBS1	miR-132-3p, miR-30a-5p, **miR-17-5p**, miR-182-5p, let-7e-5p, miR-20a
	TIMP-1	miR-26b-5p, **miR-181a-5p**, miR-29c-3p
	Col4a1	miR-29c-3p, miR-125a-5p
	EphA2	miR-26b-5p
	MMP-2	miR-17-5p, miR-29c-3p
	MMP-9	let-7e-5p
	MMP-7	miR-148a-3p
CL regression (cell death, inflamation)	IFNG	miR-7e, miR-33a
	TNF	miR-125a-5p, miR-26b-5p, miR-181a-5p
	PTGS2	miR-146a-5p
	STAT3	miR-132-3p, miR-146a-5p, miR-26b-5p, **miR-181-5p**
	SMAD4	miR-17-5p, miR-181a-5p, let-7e, miR-10a-5p, miR-20a-5p
	SMAD2	miR-17-5p, miR-1260b, miR-183-5p, miR-146a-5p, miR-26a-5p, miR-182-5p, miR-2a, miR-148a-3p
	BMPR2	miR-381-3p, **miR-17-5p**, miR-7-5p
	TGFbR2	miR-17-5p, miR-20a
	TGFbR1	miR-181a-5p
	TGFb1	miR-17-5p
	IL8	**miR-146a**, miR-17-5p, **miR-20a**
	IL18	miR-17-5p, miR-181a-5p

(a) *The candidate genes are derived from the miRTarBase, which consist of experimentally validated miRNA-target interactions. Bold: predicted targets for bovine miRNAs*.

Two miRNAs (bta-miR-2898 and −7) however, show only a trend in the regulation comparing qPCR and NGS results. This is not unusually, as different studies with human tissues have shown only partial agreement between expression data generated by different platforms (Lee et al., 2014). The trends in regulation of miR-2898 and miR-7 and corresponding significances are provided in Supplementary Figure 3.

Bta-miR-21-5p and -143, two highly abundant miRNAs counting for the separation of eCL from the other CL classes, did also exhibit the same trend of regulation comparing the qPCR and NGS results. The regulation was statistically significant between eCL and mCL for bta-miR-21-5p and between eCL and all comparisons in the case of bta-miR-143 (Tables 2, 3).

Although significant regulation could be confirmed for eight out of the ten selected miRNAs, the qPCR data show lower fold-change regulation than the fold-change regulation assessed by NGS. This is in agreement with results by different studies profiling miRNAs in tissue and plasma (Ioannidis and Donadeu, 2016, 2017).

### SOTA and unsupervised hierarchical clustering (HCL) of miRNAs

**Figure 3:**
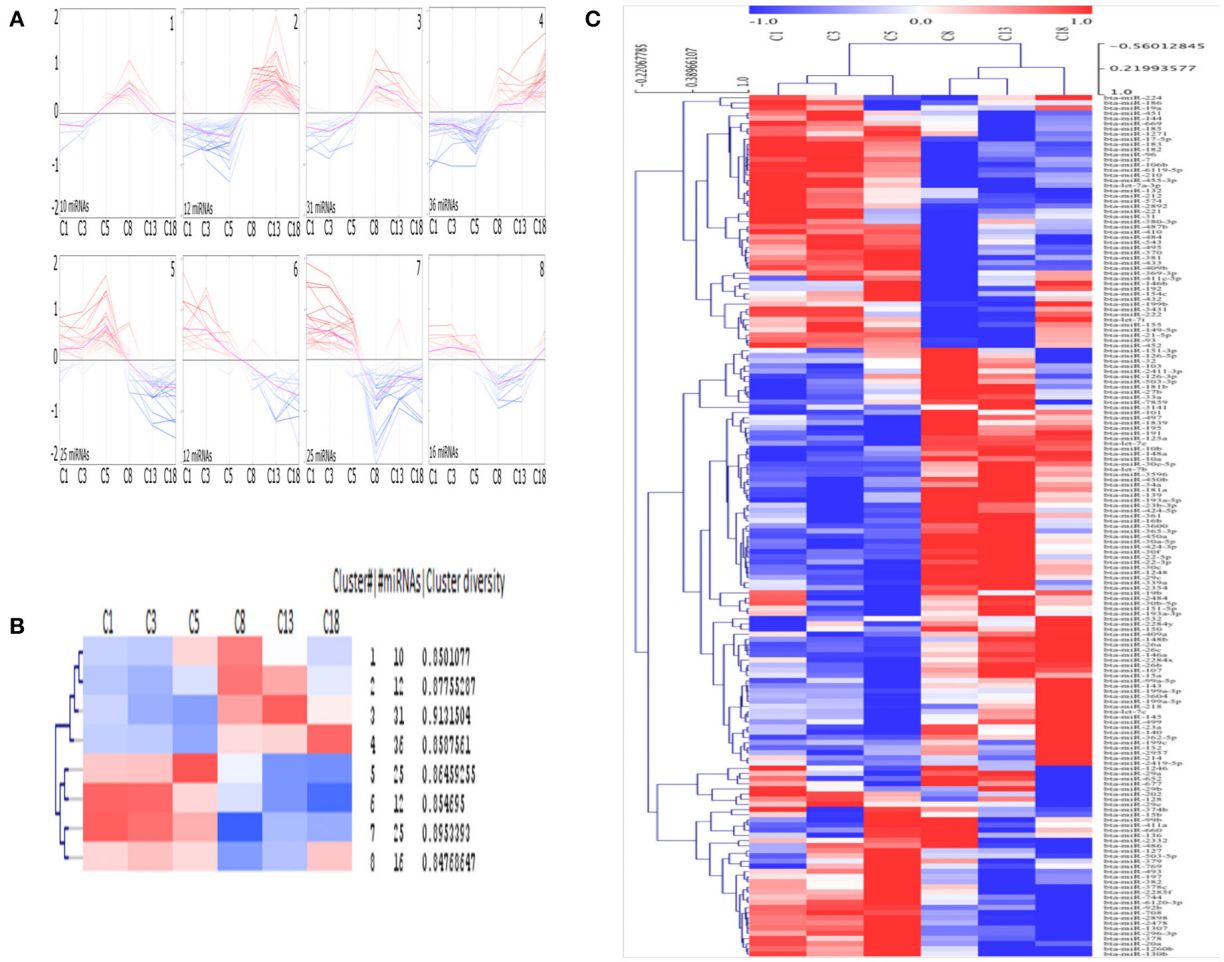
Clusters of mean centered Illumina read counts (log2 scale) with different miRNA expression profiles during early CL early CL (eCL, C1: d 1–2, C3: d 3–4, C5: d 5–7), mid CL (mCL, C8: d 8–12), late CL (lCL, C13: d 13–16), and regressed CL (rCL, C18: d > 18). **(A)** Expression graphs of all clusters, blue: down regulation, red: up regulation, pink line: mean expression. **(B)** Detailed unsupervised hierarchical clustering (HCL) dendrogram generated by Pearson Corelation Coeficient. Rows indicate single DE miRNAs and columns show CL classes across the oestrous cycle. **(C)** Dendrogram including hierarchical clustering of the eight clusters and mean expression values for each cycle stage. SOTA and HCL are performed using MeV.

Eight different clusters consisting of miRNAs with similar expression profiles were generated using SOTA (Figure 3A) and the greatest distance is calculated between the cycle stage C1 and C18. Each cluster consists of 10 to 36 miRNAs, with an average cluster diversity of 0.87 ± 0.02 (Figure 3B). According to HCL (Figure 3C, cycle time-point individually considered, see Materials and Methods section) the cycle stage C1 and C3 consist of miRNAs with similar expression pattern and the same is observed for cycle stage C8 and C13.

**Figure 4:**
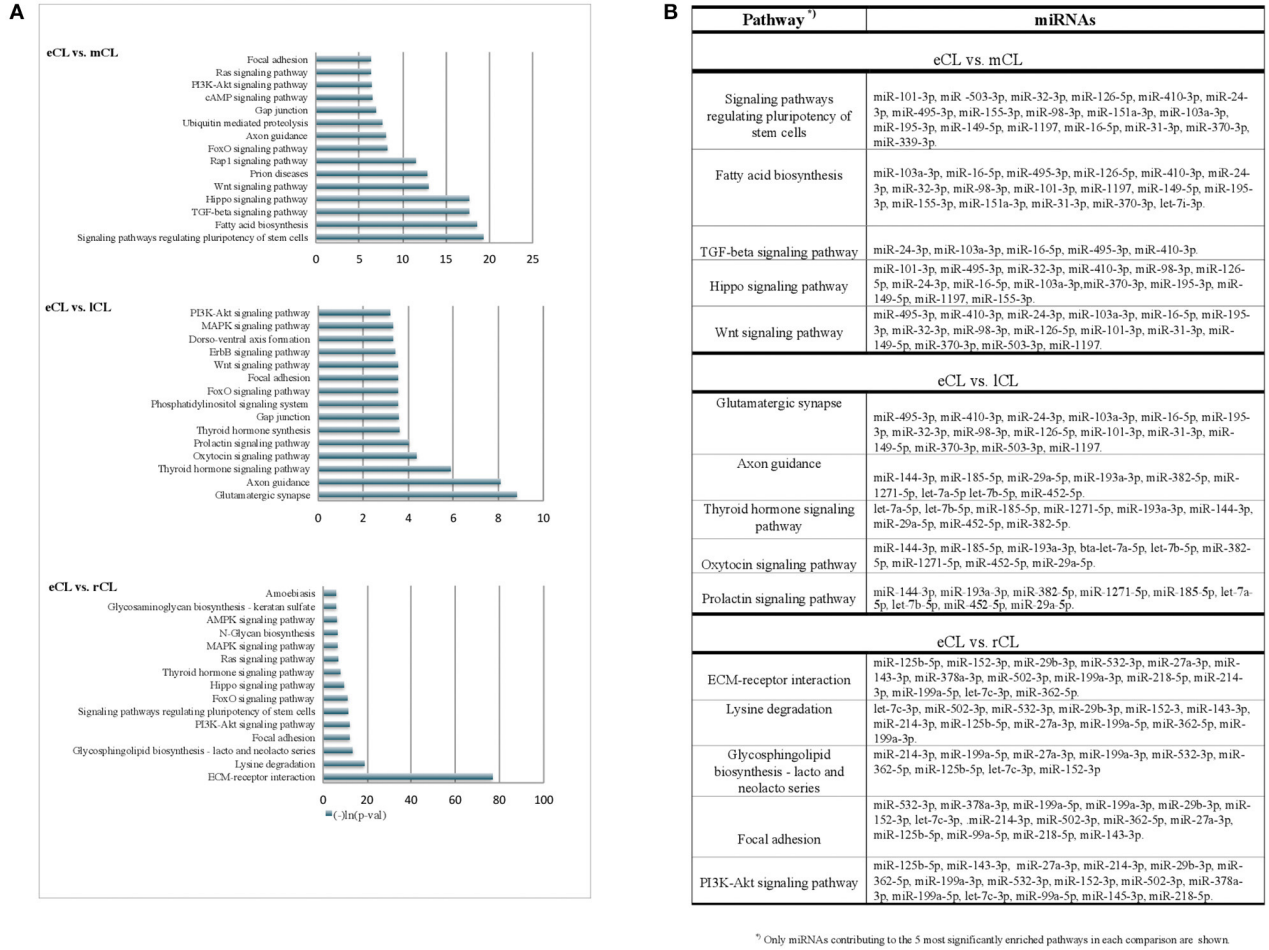
KEGG pathways enriched with genes targeted by more than 3 DE miRNAs from the comparison of individual CL classes. **(A)** Pathways enriched with genes targeted by differentially regulated miRNAs in either: eCL vs. mCL, eCL vs. lCL or eCL vs. rCL. Top 15 most significantly enriched pathways have been plotted. Lower *p*-values indicate more significance in terms of the biological function targeted by miRNAs in the pathway. **(B)** List of miRNAs that contributed to the 5 most enriched pathways between the comparisons: eCL vs. mCL, eCL vs. lCL, and eCL vs. rCL.

SOTA clusters are clearly split into two separate groups, each with an equal number of clusters. Furthermore, half of the clusters contain miRNAs with low-expression, whilst the other half contains miRNA with high-expression levels at the beginning of CL development. Again, whilst no significant changes were found during the very early CL (cycle stage: C1 and C3), for each cluster, except for cluster 1, a change in expression can be seen between samples from the cycle stage C5 (days 5–7) and those from C8 (days 8–12). This represents the time at which the CL reaches its functional state (Reynolds and Redmer, 1999). The clusters labeled 1, 2, 3, and 8 are especially interesting as the miRNAs in these clusters are primarily expressed differentially (Figure 3A) during the functional stage (cluster 1: up regulation between days 5 and 12, cluster 2 and 3: up regulation between days 8 and 12, cluster 8: down regulation between days 8 and 18 (Figures 3A,B). In contrast, miRNAs in other clusters are sequentially up or down regulated during the CL life span (cluster 4: up regulation; cluster 5, 6, 7: down regulation (Figures 4A,B). Interestingly the two clusters were observed to generally exhibit opposite expression patterns, for instance cluster 2 vs. cluster 8 and cluster 4 vs. cluster 5. The list with all individual clusters and their corresponding miRNAs can be accessed in the supplementary data (Supplementary Table 5).

### “*In-Silico*” functional annotation and pathways enrichment analysis

The search on the miRTarBase for functional annotation of the DE miRNAs resulted in the identification of numerous angiogenesis promoting genes, such as *vascular endothelial growth factor A (VEGFA), fibroblast growth factors 2 (FGF2), fibroblast growth factors 9 (FGF9), hypoxia inducible factor A (HIFA), angiopoietin 1 (ANGPT1), and angiopoietin 2 (ANGPT2)*. Furthermore, genes involved with remodeling of the extracellular matrix (ECM) were also amongst the top predicted targets of the DE miRNAs. Examples include *thrombospondin 1 (THBS1*), *tissue inhibitor of metalloproteinase 1 (TIMP-1), matrix-degrading proteinase 1 (MMP-1), and matrix-degrading proteinase 9 (MMP-9)*. Multiple cell cycle related genes were also seen as targets: *phosphatase and tensin homolog (PTEN), cyclin-dependent kinase inhibitor 1B (CDKN1B), cyclin-dependent kinase inhibitor 1A (CDKN1A), cyclin D2 (CCND2)*, and *cell division protein kinase 6 (CDK6*) as well as genes involved in the CL regression (see Table 3).

Using the DIANA tool miRPath v3.0 (Vlachos et al., 2015) with a threshold of *P* < 0.05 we searched for pathway enrichment for genes targeted by miRNA species that were unique to and DE only between individual comparisons of eCL and other three CL classes. The main molecular pathways all being those related to eCL are listed on Figure 4A (eCL vs. mCL, lCL, and rCL). The top pathways which were targeted by 3 or more unique miRNAs in the assessments between: eCL and mCL were the fatty acid biosynthesis, transforming growth factor-B (TGFB) and Wnt-signaling pathway; between: eCL and lCL, the glutamatergic synapse, axon guidance, thyroid hormone signaling, oxytocin and prolactin synthesis and between: eCL and rCL, the ECM-receptor interaction, the phosphatidylinositol 3′-kinase (*PI3K*)-Akt signaling and mitogen-activated protein kinase (MAPK)-pathway. MiRNAs that contributed to gene enrichment for the top 5 KEGG are given separately for each of the comparison in Figure 4B, whereas all significantly enriched pathways and their respective number of genes and number of miRNAs can be found in Supplementary Table 6.

## Discussion

The series of sequencing data from this study provide the most comprehensive profile of miRNAs transcriptome in bovine CL during the entire physiological estrous cycle. We used small RNA Seq (RNA Seq) to identify miRNAs expressed in the CL and found 439 miRNAs with at least one read, a number that is comparable to what other recent studies in bovine reproductive tissue have reported (Hossain et al., 2009; Maalouf et al., 2014). The identified miRNAs in this study were further annotated and grouped in two distinct clusters by principal component analysis of scores and loadings (SL-plot). The assessment of the overall data behavior (SL-plot, Figure 1B) revealed two distinct clusters, the one corresponding to the CL samples from d 1 to 7 (eCL) (SL-plot, Figure 1B, loadings: C1- C5.2) and the other to CL samples from d 8 to 12 (mCL), d 13–16 (lCL), and d > 18 (rCL). Furthermore the observed clustering of samples explained 99.8% of the variance in our data set, which we argue primarily to be composed by the different expression patterns of bta-miR-21-5p, −143, −202, −182, −96, −181, −10a, and −146a (SL-plot, Figure 1B, Scores).

Irrespective of the cycle stage we found a number of miRNAs to be highly expressed in the CL throughout its lifespan (Figures 1C–F). In total, 22 miRNAs (bta-miR-21-5p, -miR-143, -miR-10b, -let-7i, -miR-202, -miR-148a, -let-7f, -miR-3600, -miR-99a55p, -let-7a-5p, -miR-27b, -miT-100, -let7g, -miR-26a, -miR-378, -miR-30d, -miR-125b, -450a, -miR-30e-5p, -let-7b, -miR-199a-3p, and -miR-26c) contributed to the top twenty most abundantly sequenced miRNAs in the bovine CL. Each of these miRNAs was also significantly DE in at least one of the individual comparisons between the eCL and three other CL classes (Supplementary Table 3). MiRNA sequences, which align with and hence correspond to bta-miR-21-5p and bta-miR-143-3p account for >40% of the overall reads (Supplementary Table 2). Moreover, they show opposing and significantly DE at specific CL classes, namely bta-miR-21-5p is significantly upregulated in eCL relative to mCL and lCL (FC = 1.078 and adjusted *P* = 6.84E-04; FC = 1.119 and adjusted *P* = 2.71E-04, Supplementary Table 3, eCL vs. mCL, eCL vs. lCL), while bta-miR-143 is significantly downregulated in the eCL vs. the rCL (FC = −1.15 and adjusted P = 3.04E-06, Supplementary Table 3 eCL vs. rCL).

A network of forty-two miRNAs (Figures 2B,C) was identified as consistently and significantly DE in the eCL class relative to their expression at the mCL, lCL, and rCL. Of them five miRNAs (bta-miR-10b, -miR-26a, -miR-30a-5p, -miR-450a, and -miR-202) were as well-classified among the top 20 most abundantly expressed miRNAs. The remaining miRNAs showed sequentially up- or downregulation in eCL vs. mCL, lCL, and rCL classes (Table 1). However seven miRNAs (bta-miR-210-3p, −2898, −96, −7-5p, −183-5p, −182, and −202) show drastic up-regulation with a FC ≥ 2.0 between the eCL and the three other CL classes, while bta-miR-146a is the only miRNA found to be differentially downregulated at the same extent in the eCL vs. lCL and rCL (Figures 2B,C, miRNAs with FC ≥ 2.0 and adjusted *P* < 0.01 are indicated with asterisks). Furthermore all miRNAs were validated by RT-qPCR and the trend of regulation was significant for five of them, while bta-miR-2898 and bta-miR-7 show only a trend in regulation (Table 3, Supplementary Figure 3).

The identification of bta-miR-183-5p, -96, and -182 with increased expression in the eCL vs. the other three cycle stages (Figures 2B,C) is consistent with results from previous studies in bovine (Sontakke et al., 2014; Gebremedhn et al., 2016). All three miRNAs are shown to regulate bovine granulosa cell proliferation by targeting *FOXO1* (Gebremedhn et al., 2016). Although, miR-96 was experimentally validated to also target *FOXO1* both in human (Guttilla and White, 2009) and bovine (Gebremedhn et al., 2016), its role in bovine CL (luteal cells) has been partially explained. Using human luteinized granulosa cells (hLGC) and bovine CL from day 1 to 4 of the cycle, (Mohammed et al., 2017) identified miR-96 as the key regulator of luteal cell survival by modulating the effect of FOXO1 on steroidogenesis (P4). However, in contrast to hLGC they could not show an inhibitory effect for miR-96 on P4 production in bovine CL. Emphasizing thus the limitation in miR-target interaction validation when using a cross-species approach.

Our sequencing results provide the expression pattern for all three mRNAs: miR-96, -183-5p, and -182 in bovine CL also throughout the cycle and not only for the very early days of the cycle (d 1–4). Interestingly, we found all three miRNAs to peak in their number of reads in the eCL followed by a significant down-regulation in mCL and further steadily up-regulation in lCL and rCL (Table 1). This observation suggests miR-96, -183-5p, and -182 to be involved not only during very early CL development in the process of follicle-luteal transition as suggested by Gebremedhn et al. (2016), but also in the steps thereafter when the CL becomes functional (mCL and lCL) and then regresses (rCL). Indeed, a sustained P4 production is required throughout the functional stage of the CL (eCL, mCL, and lCL) and in contrast a decline in P4 production signals a regressing CL (Davis and Rueda, 2002). Since these three miRNAs belong to the same cluster and exhibit similar expression dynamic in bovine CL stages analyzed in this study (Table 1) they presumable regulate other target genes than FOXO1 coordinately to ensure P4 synthesis in the subsequent sates.

Further, we identified bta-miR-202 not only as highly abundant in eCL, mCL, and lCL (Figures 1C–E) but also drastically upregulated in eCL vs. the rCL (Table 1, FC = 2.3 and adjusted *P* = 3.88E-10; Table 3, RT-qPCR FC = 0.2 and *P* = 0.0015). Sontakke et al. (2014), provide the first evidence for the involvement of bta-miR-202 in bovine reproductive tissue and show that bta-miR-202 regulates the viability of mural follicles. Notably, they did a body-wide screening that revealed bta-miR-202 to be expressed exclusively in the gonads. Studies in human have linked mir-202 with different types of cancer acting thus as tumor suppressor or oncogene (reviewed by Lee and Dutta, 2009). In line with the physiology of regressing CL, which is characterized by an enhanced cell death (Stocco et al., 2007) and in contrast to eCL where cell death most be prevented (Sugino and Okuda, 2007), bta-miR-202 peaks in its number of reads in the eCL and the reads fall drastically in the regressing CL (Table 1, DESeq2 normalized reads in eCL = 31′881; reads in rCL = 5′617). The role of bta-miR-202 in the bovine CL is not known, however in the eCL development bta-miR-202 might be acting in similar manner as in cancers: to block cell proliferation, invasion, or apoptosis.

The expression data generated by sequencing and further confirmed by RT-qPCR (Tables 2, 3 and Supplementary Figure 3) highlights for the first time the dynamic regulation of miRNAs over the time of the estrous cycle in bovine CL. Furthermore our results are consistent with findings from profiling studies of circulating miRNAs during the bovine estrous cycle and pregnancy (Ioannidis and Donadeu, 2016, 2017). Considering that CL's functionality is critical for a physiological cycle and a successful pregnancy, the identification of circulating miRNAs originating from the CL is of particular importance. Recently Ioannidis and Donadeu (2017) identified changes in circulating miRNAs as early as day 8 of the pregnancy in cattle and provide important candidate biomarker for early pregnancy detection. Bta-miR-26a and bta-miR-143, among others were identified as potential biomarkers. Intriguing, bta-miR-143 was shown to be the only miR found in circulation but not originating from the blood, thus letting us hypotheses it could be of endocrine origin and is secreted from the CL. Future studies profiling at the same time circulating miRNA and miRNAs from the CL will facilitated biomarker identification important for early CL function and other CL functional stages.

With regard to the most abundantly expressed miRNAs bta-miR-21-5p is of particular interest since many studies have demonstrated both its up-regulation in response to postovulatory LH surge (Fiedler et al., 2008), as well as its regulatory effect on different apoptosis genes to promote cell survival (Asangani et al., 2008; Carletti et al., 2010; Buscaglia and Li, 2011). Survival of the ovarian granulosa cells is decisive for their transition into luteal cells and along with the process of follicle basal membrane cells degradation it enables angiogenesis and early CL development (Reynolds and Redmer, 1999; Skarzynski et al., 2013). The cellular regulation of both of these events corresponds to the highest expression level of bta-miR-21-5p, specifically in the early CL (Figure 1C, DESeq2 normalized reads = 320 × 10^3^). This observation and published data from mice studies (Fiedler et al., 2008; Carletti et al., 2010) let us to hypothesize bta-miR-21-5p had an indirect effect on bovine eCL development. Initially, it is the inflammatory like response elicited after the LH (Richards et al., 2008) surge that is shown to increase miR-21 expression from pre-miR-21 via the *IL6* and transcription factor *STAT3* (Löffler et al., 2007; Han et al., 2012) and following its abundance that facilitates angiogenesis via the suppression of the reversion-inducing, cysteine-rich protein with kazal motifs (*RECK)* gene, which is shown to negatively regulate transcription of *MMP1* and *MMP9* (Takagi et al., 2009; Han et al., 2012). *MMP*s activity is required for cellular degradation of basal membrane prior to angiogenesis (Oh et al., 2001). Both of these *MMPs* are shown by our group (Kliem et al., 2007) to be highly expressed in bovine in the eCL (d 1–7) and mid CL (d 8–12). Accordingly, we propose that the increase in *STAT3* mediated bta-miR-21-5p expression leads to downregulation of apoptosis genes (Buscaglia and Li, 2011) and the *RECK* mRNA levels, allowing thus granulose cell transition in luteal cells and *MMPs* to be active for the degradation of basal membrane cells and propagation of new blood vessel form existing ones (angiogenesis). Furthermore, we identified bta-miR-146a as drastically downregulated in eCL vs. the mCL and lCL. Bta-miR-146a has just recently been shown to act as a negative feedback regulator of bovine inflammation and innate immunity through downregulation of the TLR4/TRAF6/NF-κB pathway (Wang et al., 2017) and we propose bta-miR-146a supports the activity of bta-miR-21-5p by downregulating the post-ovulation inflammatory mediators such as *IL6* and *IL8* in the early CL in bovine.

Bta-miR-7-5p and bta-miR-2898 are also identified as drastically upregulated in eCL vs. the mCL and lCL, rCL respectively (Figures 2B,C). Studies implicating bta-miR-7-5p in bovine are limited to its presence and identification in immune-related tissues (Coutinho et al., 2007) and to date no functional validation studies are performed in bovine or other species. Whereas, recent studies show bta-miR-2898 expression to be upregulated in mammary gland tissues of mastitis-infected cows (Wang et al., 2014). This implicates bta-miR-2898 in the immune response relevant to mastitis, however in the miRTarBase experimentally validated interactions for this miRNA are not provided. Consequently its role in bovine CL needs further investigation.

CL formation has similarities to the processes involved with wound healing and tumorogenesis, both of which that are enabled by extensive angiogenesis (Niswender et al., 2000). Interestingly, in mice miR-21 has also been shown to induce tumor angiogenesis by targeting *PTEN* and activating AKT and ERK1/2 signaling pathways, which then in turn enhances *VEGF* and *hypoxia-inducible factor 1-alpha (HIF1A)* expression (Liu et al., 2011). Our group showed that in bovine CL, the highest expression of *VEGF* and *VEGFR-2* is detected during days 1–7 (eCL) of the estrous cycle (Berisha et al., 2000b, 2017 unpublished data) and that is concurrent with increased bta-miR-21-5p expression we observed in the eCL (Figure 1C, d 1–7). Thus, similar to studies with mice (Liu et al., 2011) and because of its high abundance in the eCL, bta-miR-21-5p might activate AKT and ERK1/2 signaling pathways in bovine CL and promote angiogenesis through enhancement of *VEGF* expression at that time.

In contrast, the elevated expression of *HIF1A*, which has been documented by our group to be the highest in bovine CL during d 1–7 (Berisha et al., 2017) may in turn regulate the expression of other miRNAs and likely supports the activity of bta-miR-21-5p. In agreement, bta-miR-210-3p one of the drastically upregulated miRNA in the eCL vs. mCL (FC = 2.4, adjusted *P* = 2.26E-16, Figure 2B, Table 1), was shown to be induced under hypoxic conditions (Ivan and Huang, 2014). Hypoxic conditioning is documented in the early CL of monkeys (Tesone et al., 2005) and hypoxia is critical for establishing vascularization in bovine CL (Nishimura and Okuda, 2010). Furthermore, *HIF1A* is an experimentally validated target gene for human micro-210-3p (hsa-mir-210-3p) (Table 3), but not for bta-miR-210-3p (http://www.targetscan.org). Nevertheless, *HIF1A* is an experimentally validated target for has-miR-17-5p and -20a (http://mirtarbase.mbc.nctu.edu.tw/) and is also successfully *in silico* predicted for bta-miR-17-5p and -20a (http://www.targetscan.org). Notable, bta-miR-17-5p and bta-miR-20a are DE miRNAs identified in our study and show a FC < 2.0 (Table 1).

Around day 8 of the cycle, the hormonal environment becomes optimal and the CL produces P4 at levels sufficient to maintain the cellular phenotype at the mid CL stage (Wiltbank, 1994). However, shortly after this and in the absence of pregnancy the CL becomes responsive to *prostaglandin F2 alpha (PGF2A)* and starts regressing (Skarzynski et al., 2013). The regression of bovine CL is a complex process that starts with a decline in the synthesis of P4 (functional luteolysis), followed by tissue involution and cell death (Berisha et al., 2010). It involves multiple different factors, including *IFNG, TNF, ANGPT2, IGF1, STAT3, SMAD2/4, TGFb1, IL8*, and *IL18* (Sugino and Okuda, 2007; Hou et al., 2008; Berisha et al., 2010). Most of these factors are experimentally validated gene targets for human miRNAs (Table 3), however very few are also *in silico* predicted for bovine miRNAs (Table 3, miRNAs in bold).

The regression of CL is accompanied by extensive extracellular matrix remodeling and deposition of *collagen type I (COL1A1*) (Irving-Rodgers et al., 2006). Regression of the CL is likely to be facilitated by the second most abundantly expressed miRNA identified in this study bta-miR-143, which is significantly upregulated in rCL relative to the eCL (Figure 1F, Supplementary Table 3C) and *COL1A1* is an experimentally validated target gene for has-miR-143 and successfully predicted for bta-miR-143 (Table 3). Further miR-143 targets genes related to P4 synthesis and cell death including *COX2, IGF1R, IFNG, TNFA, and Bcl-2* (Table 3). These genes are relevant for CL regression, as the abundance of most of them is altered upon PGF2A administration. Some of them, specifically *TNFA*, act as intra-ovary factors and induce apoptotic death by modulating the *Bcl-2* family of genes, and caspase 3 (Skarzynski et al., 2005; Kliem et al., 2009). Furthermore miR-143 has been shown to promote porcine granulose cell apoptosis and follicular atresia by suppression the *follicle stimulating hormone receptor (FSHR)* (Du et al., 2016).

Another interesting finding of this study follows from the self-organizing tree analysis (SOTA). Using SOTA we showed that differentially expressed miRNAs across the 21 day cycle demonstrate a specific pattern of expression. Based on a correlation of their expression, miRNAs were grouped in eight distinct clusters (Figures 3A,B). The clusters themselves were furthermore clearly split into two groups, one consisting of the miRNAs upregulated at the beginning of the cycle (Figure 3A, bottom 4 panel), and the other of miRNAs upregulated in the mid-, late-, and regressed CL stages (Figure 3A, top 4 panel). We theorize that this observation is due to “progesterone induced miRNAs expression.” MiRNAs in cluster 7 (miR-29b, −31, −210, and let-7a) for instance are shown to be increased with progestin in human glandular epithelium derived from the uterus (Kuokkanen et al., 2010). However, despite this, to date no progestin response elements have been identified in the promoter region of miRNAs shown to be regulated by progestin. Therefore, the data should be viewed with this in mind and also by considering the other non-genomic means (Kuse et al., 2015), by which P4 can induce miRNA expression and may also be considered as determining factor for the cycle stage dependent miRNA expression. Notable examples of this include miRNAs having been shown to stably circulate in the blood in exosomes and therefore that they may also regulate gene expression in a paracrine manner (Zhang et al., 2015). There is much to be discovered about this exciting mechanism but it may represent one mode of action for miRNAs in the regulation of this dynamic gland.

The miRNAs we identified as differentially expressed only between individual comparisons of eCL to mCL are likely targeting genes involved in fatty acid biosynthesis, as well as TGFB-, Wnt-, and FoxO- signaling (Figure 4A). Further, pathways enriched with genes targeted by miRNA DE between only between eCL and lCL included the glutamatergic synapse, axon guidance, thyroid hormone signaling pathway, oxytocin and prolactin signaling (Figure 4B). Pathway enrichment for genes targeted by miRNAs DE only between eCL and rCL revealed the ECM-receptor interaction as the most enriched pathway, followed by lysine degradation, glycosphingolipid biosynthesis—lacto and neolacto series and the focal adhesion signaling pathway (Figure 4C). Which individual miRNAs contributed to pathway enrichment is accessible as additional file to this paper.

Overall, the pathways we see to be enriched for genes targeted by miRNA DE only between individual compression are known to be involved in the early luteal CL development, formation and luteinisation of CL.

## Conclusion

This study utilizes a small RNA sequencing technology to temporally profile miRNA transcriptome in the CL from cycling cows. It is the first study, to our knowledge, to analyze the dynamics of miRNAs expression at six different timely defined CL classes, throughout the entire 21 days of the bovine estrous cycle.

It enabled the identification of bta-miR-21-5p and bta-miR-143 as two species that were highly abundant regardless of CL developmental or functional stage. We demonstrated that 42 miRNAs are consistent significantly different expressed at mCL, lCL, and rCL compared to their expression in the eCL. Out of them bta-miR-210-3p, -2898, -96, -7-5p, -183-5p, -182, and -202 show drastic up-regulation between the eCL and three subsequent CL classes, while bta-miR-146a is the only miRNA found to be differentially downregulated at the same extent in the eCL vs. lCL and rCL. These miRNAs have predicted targets involved in regulating critical angiogenic and antiangiogenic, as well as luteotropic and luteolytic genes.

In addition, we identified groups of miRNAs DE only at individual comparison's: 24 miRNAs in eCL vs. mCL only, 11 miRNAs in eCL vs. lCL only and 21 miRNAs in eCL vs. rCL only. Each of these groups of DE miRNAs was found to regulate distinct pathways involved in the progression of the CL from early development, to fully functional and regressing phase.

Using the self-organizing tree algorithm (SOTA) we grouped all DE miRNAs into eight clusters based on their expression profiles. These clusters were clearly split into two larger groups, consisting of miRNAs up regulated at either the beginning of the cycle or the mid-, late- and regressed stages of the CL cycle.

Considering the very limited access to healthy human CL tissue for research reasons, the similarities between early CL development and tumorogensis; and the similarity in ovarian physiology between cattle and human, this study is of particular importance and provides novel insights in the dynamics of miRNA transcriptome during the physiological estrous cycle.

## Availability of data and materials

The sequencings files are deposited at the ENA database and are available under the accession number: PRJEB20028.

## Author contributions

RMG and BB: Conceived of the study and participated in its design; RMG: Wrote the manuscript. RMG and CIS: Performed the library preparation for Illumina HiSeq2500 sequencing platform; BK, RMG, and CIS: Conducted statistical analysis; BB, MWP, IR, and RT-C: Reviewed the manuscript. All authors read and approved the final manuscript.

### Conflict of interest statement

The author IR was employed by the company Eurofins Medigenomix Forensik GmbH. The other authors declare that the research was conducted in the absence of any commercial or financial relationships that could be construed as a potential conflict of interest.
