# Supplementary material for: The Dynamics of microRNA Transcriptome in Bovine Corpus Luteum during Its Formation, Function, and Regression
Source: Front Genet. 2017 Dec 15;8:213. doi: 10.3389/fgene.2017.00213 (PMC5736867; doi:10.3389/fgene.2017.00213)
Supplement: Supplementary file 1 [file Table1.DOCX]

|  | C1 | C3 | C5 | C8 | C13 | C18 | Mean | SD |
| --- | --- | --- | --- | --- | --- | --- | --- | --- |
| Total sequences | 15490030 | 13269984 | 16796297 | 23203853 | 14999002 | 11339724 | 15244516 | 4070073 |
| Passed trimming >16 nts | 9406859 | 8438448 | 10422350 | 13456230 | 9016777 | 7646058 | 9211818 | 2048812 |
| Failed trimming < 16nts | 6082264 | 4830594 | 6372898 | 9746356 | 5981294 | 3692932 | 6031779 | 2039033 |
| * RNAcentral filtered | 3718275 | 3648571 | 3846753 | 4907054 | 3244713 | 3027548 | 3683423 | 653417 |
| RNAcentral unmapped | 5688583 | 4789876 | 6575597 | 8549176 | 5772064 | 4618511 | 5730324 | 1438850 |
| Annotated reads miRBase bta | 624997 | 594773 | 762278 | 1262728 | 1035079 | 1185501 | 898679 | 289266 |
| % (/total sequences) bta | 4.1% | 4.3% | 4.8% | 5.5% | 7.0% | 11.8% | 5.1% | 2.9% |
| Unmapped reads miRBase bta | 5063587 | 4195104 | 5813319 | 7286448 | 4736984 | 3433010 | 4900285 | 1343081 |
| Annotated reads miRBase hsa | 429277 | 403509 | 436703 | 841830 | 818620 | 886245 | 627662 | 234454 |
| % (/total sequences) hsa | 2.8% | 2.9% | 2.6% | 3.7% | 5.5% | 8.7% | 3.3% | 2.4% |
| Unmapped reads miRBase hsa | 5259306 | 4386367 | 6138894 | 7707346 | 4953443 | 3732266 | 5106375 | 1406383 |

* RNAcentral: known small RNAs like rRNA, snRNA and tRNA. Average for n=3 for each CL class (C1- C18) is presented.

**Table 1.** Evaluation of sequencing data, after data trimming and genome mapping: the mean total number of sequences was 15,244,516, of which 898,679 are annotated reads that account for 5.1% of the sequence total.
