# Supplementary material for: The Dynamics of microRNA Transcriptome in Bovine Corpus Luteum during Its Formation, Function, and Regression
Source: Front Genet. 2017 Dec 15;8:213. doi: 10.3389/fgene.2017.00213 (PMC5736867; doi:10.3389/fgene.2017.00213)
Supplement: Supplementary file 7 [file Image1.PDF]

A)

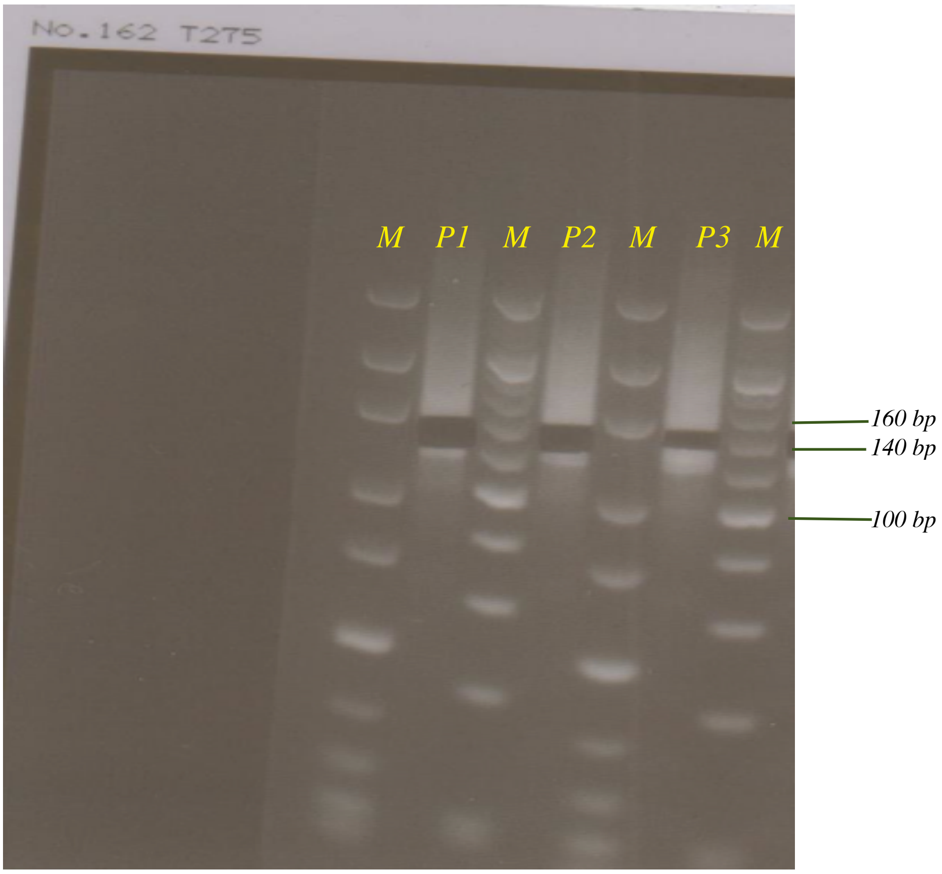

B)

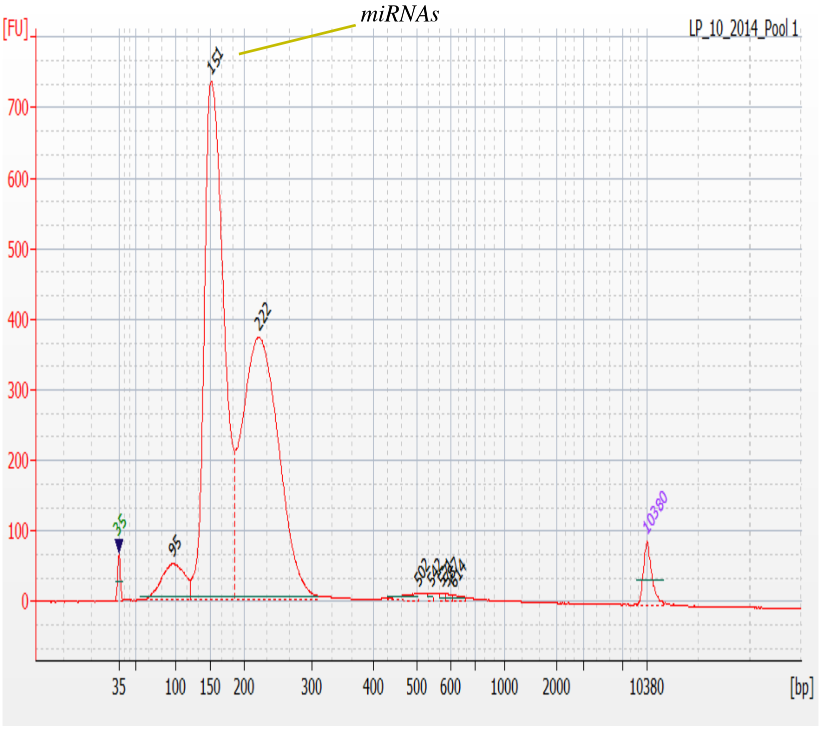

Libraries are generated with NEBNext Multiplex Small RNA Library Prep Set for Illumina.
A) cDNA constructs were isolated by loading the PCR products on a 4% agarose gel.
Bands corresponding to products of 140-160 bp in length were cut, purified and sequenced on the Illumina HiSeq2500 platform.
M: marker, P1- P3: pooled small RNA libraries.
B) The size and quantity of the cDNA library was validated using the High Sensitivity DNA Kit (Agilent Technologies, Germany).
