# Supplementary material for: The Dynamics of microRNA Transcriptome in Bovine Corpus Luteum during Its Formation, Function, and Regression
Source: Front Genet. 2017 Dec 15;8:213. doi: 10.3389/fgene.2017.00213 (PMC5736867; doi:10.3389/fgene.2017.00213)
Supplement: Supplementary file 8 [file Image2.PDF]

A)

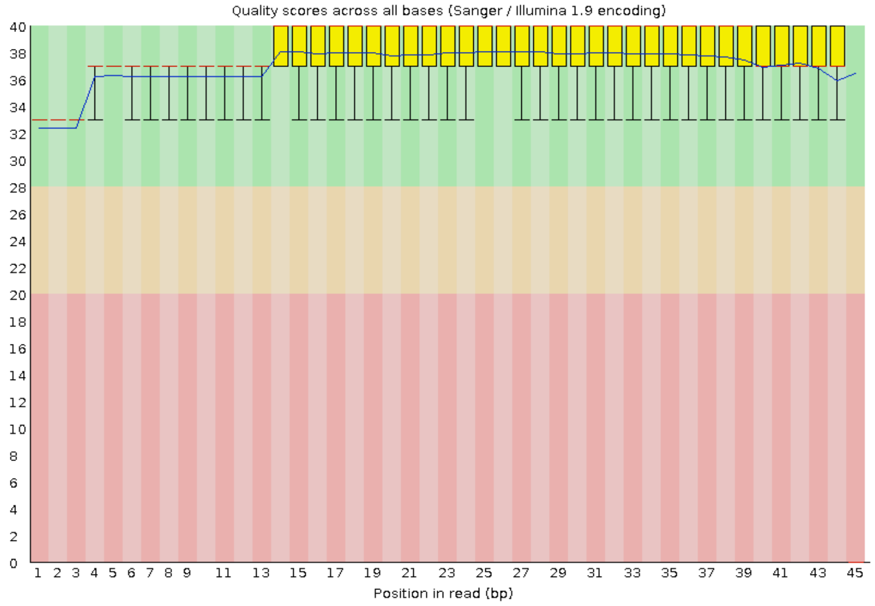

B)

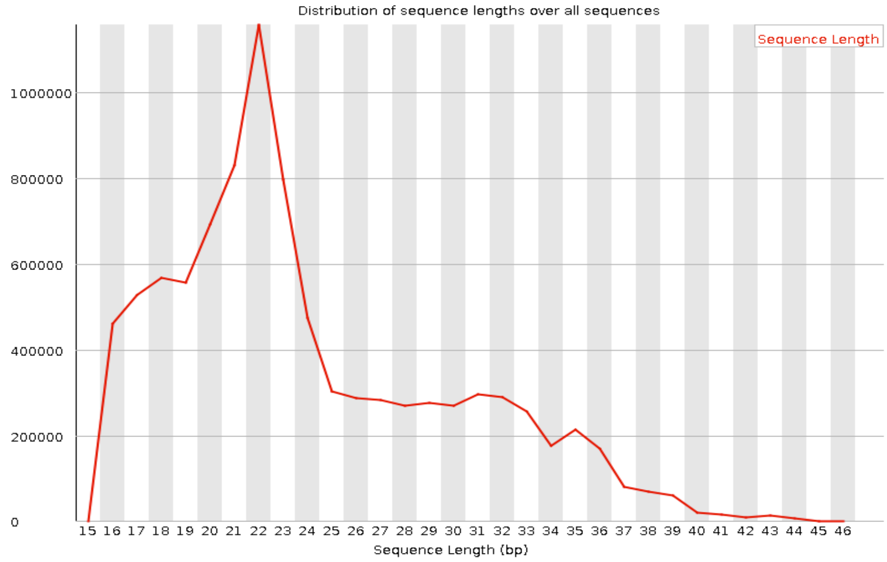

C)

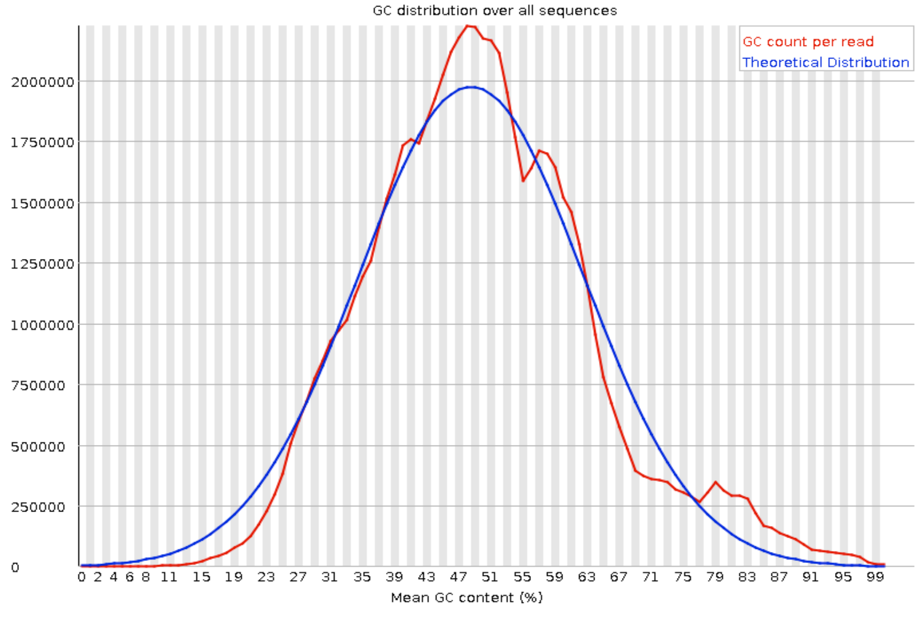

A) Overall, 98% of all reads showed a quality (Q) score of 37.
B) The total sequence length showed was 22 nts and the
C) mean GC content per sequence was around 47%.
