# Supplementary material for: The Dynamics of microRNA Transcriptome in Bovine Corpus Luteum during Its Formation, Function, and Regression
Source: Front Genet. 2017 Dec 15;8:213. doi: 10.3389/fgene.2017.00213 (PMC5736867; doi:10.3389/fgene.2017.00213)
Supplement: Supplementary file 9 [file Image3.PDF]

Trends in the regulation of DE miRNAs: RT-qPCR and NGS data expression

A)

miRNAs	qPCR	NGS	SEM (qPCR)	SEM (NGS)
	**Fold-change: eCL / mCL**			
bta-miR-210	0.3	2.4	0.17	0.27
bta-miR-96	0.2	2.8	0.28	0.45
bta-miR-7	0.5	2.2	0.40	0.21
bta-miR-182	0.2	2.6	0.17	0.44
bta-miR-183	0.3	2.5	0.27	0.31
bta-miR-2898	1.0	1.6	0.09	0.40
bta-miR-146a	-2.0	-1.5	0.19	0.24
bta-miR-202	0.6	1.3	0.06	0.33
bta-mir-21-5p	0.48	1.08	0.30	0.27
bta-miR-143	-2.41	-0.51	0.83	0.22

B)

miRNAs	qPCR	NGS	SEM (qPCR)	SEM (NGS)
	**Fold-change: eCL / lCL**			
bta-miR-210	0.8	1.7	0.25	0.27
bta-miR-96	0.3	2.1	0.05	0.45
bta-miR-7	1.2	1.5	0.55	0.21
bta-miR-182	0.5	1.7	0.09	0.43
bta-miR-183	0.5	1.8	0.09	0.40
bta-miR-2898	0.7	2.3	0.09	0.31
bta-miR-146a	-4.4	-2.2	0.86	0.24
bta-miR-202	1.2	1.0	0.08	0.33
bta-mir-21-5p	0.9	1.1	0.25	0.27
bta-miR-143	-3.9	-0.5	0.71	0.22

C)

miRNAs	qPCR	NGS	SEM (qPCR)	SEM (NGS)
	**Fold-change: eCL / rCL**			
bta-miR-210	0.6	1.4	0.16	0.27
bta-miR-96	0.1	2.3	0.09	0.45
bta-miR-7	0.7	1.2	0.31	0.21
bta-miR-182	0.2	2.1	0.15	0.44
bta-miR-183	0.3	2.2	0.25	0.40
bta-miR-2898	0.7	2.5	0.36	0.31
bta-miR-146a	-4.3	-2.6	1.37	0.24
bta-miR-202	0.2	2.3	0.00	0.33
bta-mir-21-5p	1.07	0.13	0.14	0.27
bta-miR-143	-3.72	-1.16	1.36	0.22

Trends in the regulation of DE miRNAs: RT-qPCR and NGS data expression

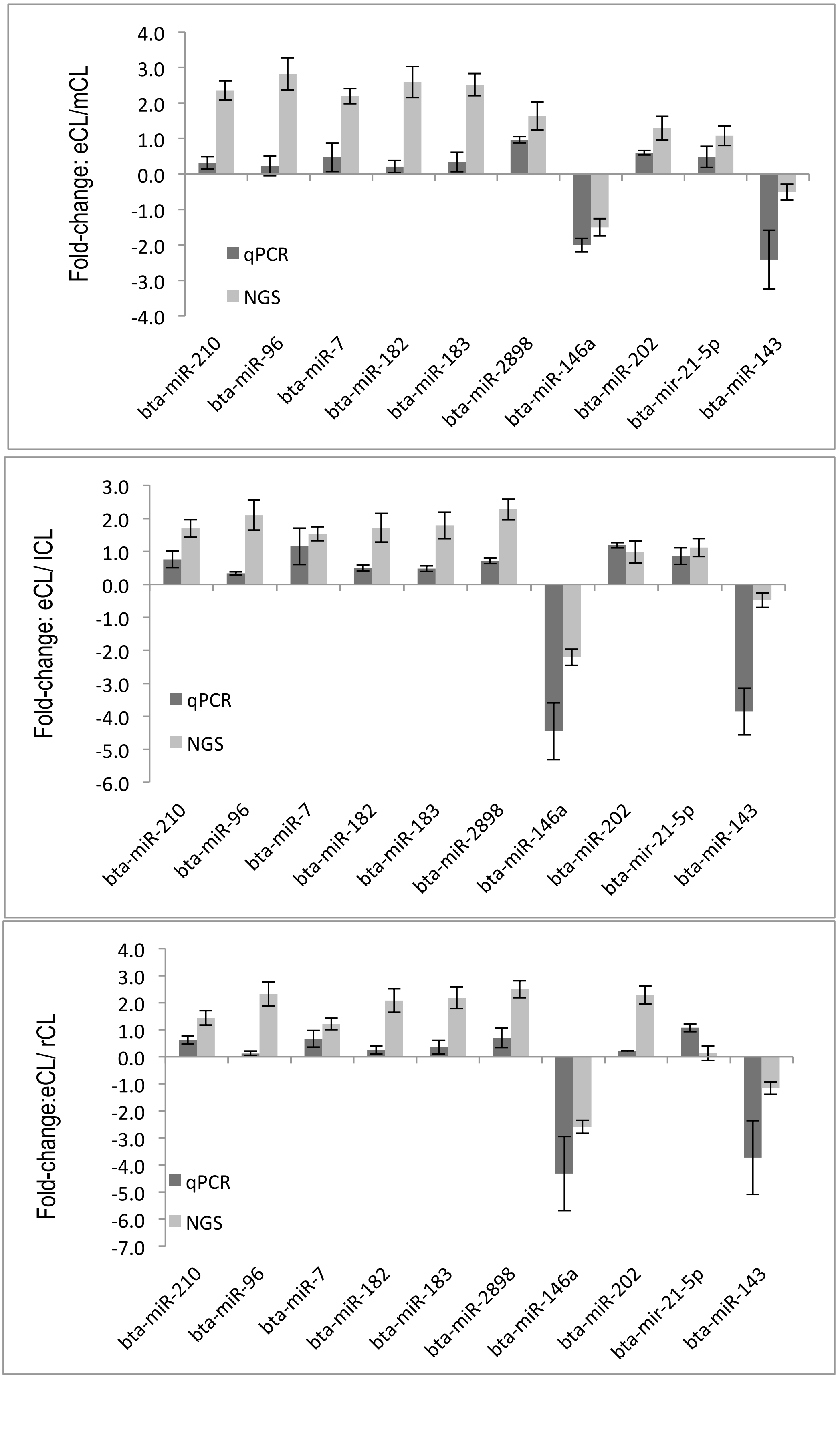

RT-qPCR validation and significance for individual miRNAs

		eCl/mCL	eCL/lCL	eCL/rCL
**Bta-miR-210**	2^(-ddCt)	0.48	0.76	0.62
	SEM	0.173	0.254	0.156
	ttest	**0.030**	0.266	0.117

		eCl/mCL	eCL/lCL	eCL/rCL
**Bta-miR-96**	2^(-ddCt)	0.22918	0.33635	0.12061513
	SEM	0.2773174	0.0461274	0.08834827
	ttest	**0.0012887**	**0.0079184**	**0.00144017**

		eCl/mCL	eCL/lCL	eCL/rCL
**Bta-miR-7**	2^(-ddCt)	0.4709246	1.1552158	0.6632465
	SEM	0.401942	0.5517755	0.30819771
	ttest	0.5127386	0.0777077	0.88958993

		eCl/mCL	eCL/lCL	eCL/rCL
**Bta-miR-182**	2^(-ddCt)	0.2093772	0.5005105	0.24738237
	SEM	0.1698101	0.0929313	0.14781098
	ttest	**0.0005629**	**0.0495864**	**0.0077687**

		eCl/mCL	eCL/lCL	eCL/rCL
**Bta-miR-183**	2^(-ddCt)	0.3369091	0.4783102	0.34842051
	SEM	0.2724848	0.0866872	0.25246713
	ttest	**0.0416063**	0.1545229	**0.02713599**

		eCl/mCL	eCL/lCL	eCL/rCL
**Bta-miR-2898**	2^(-ddCt)	0.9646612	0.7173355	0.69921464
	SEM	0.0888755	0.0856588	0.35756479
	ttest	0.7844567	0.0659098	0.065358

		eCl/mCL	eCL/lCL	eCL/rCL
**Bta-miR-146a**	2^(-ddCt)	2.0022167	4.4485925	4.31313449
	SEM	0.1927118	0.8595513	1.37366551
	ttest	0.064268	**0.0018543**	**0.00770503**

		eCl/mCL	eCL/lCL	eCL/rCL
**Bta-miR-202**	2^(-ddCt)	0.5987793	1.1902033	0.22199682
	SEM	0.0614416	0.0794587	0.14911455
	ttest	0.0579422	0.4982357	**0.00153976**

RT-qPCR validation and significance for individual miRNAs

		eCl/mCL	eCL/lCL	eCL/rCL
**Bta-miR-21-5p**	2^(-ddCt)	0.48221103	0.86017904	1.073600141
	SEM	0.2964109	0.25170561	0.144794741
	ttest	0.46763485	0.86463907	**0.07215558**

		eCl/mCL	eCL/lCL	eCL/rCL
**Bta-miR-143**	2^(-ddCt)	2.41209959	3.85416062	3.723778331
	SEM	0.82912227	0.7053813	1.363012553
	ttest	**0.02416748**	**0.00028332**	**0.00151253**
